# A Biological Transition Around 200 × 10^3^ Somatic Cells/mL in Dairy Cows: Inflammatory and Oxidative Changes

**DOI:** 10.3390/ijms27177845

**Published:** 2026-09-02

**Authors:** Jan Slósarz, Marek Balcerak, Grzegorz Grodkowski, Paweł Solarczyk, Piotr Kostusiak, Małgorzata Kunowska-Slósarz, Aleksandra Kalińska, Kinga Grodkowska, Kamila Puppel

**Affiliations:** Institute of Animal Science, Warsaw University of Life Sciences, Ciszewskiego 8, 02-786 Warsaw, Poland; jan_slosarz@sggw.edu.pl (J.S.); marek_balcerak@sggw.edu.pl (M.B.); grzegorz_grodkowski@sggw.edu.pl (G.G.); pawel_solarczyk@sggw.edu.pl (P.S.); piotr_kostusiak@sggw.edu.pl (P.K.); malgorzata_kunowska-slosarz@sggw.edu.pl (M.K.-S.); aleksandra_kalinska@sggw.edu.pl (A.K.); kinga_grodkowska@sggw.edu.pl (K.G.)

**Keywords:** mammary gland, cytokines, IL-8, innate defense, redox homeostasis, metabolic status, segmented regression, change-point analysis

## Abstract

Somatic cell count (SCC) is routinely used as an indicator of mammary gland health, but the SCC level at which coordinated inflammatory and systemic changes become apparent remains uncertain. This study aimed to characterize biological changes across increasing SCC and identify the SCC range associated with a coordinated transition. The study included 580 clinically healthy Polish Holstein–Friesian cows without detectable intramammary infection (IMI), classified according to milk SCC as <100, 100–199, 200–399, or ≥400 × 10^3^ cells/mL. Milk was analyzed for composition, inflammatory cytokines, and mammary innate-defense proteins, while blood was assessed for metabolic and hepatic indicators and oxidative-status markers, including malondialdehyde (MDA), total antioxidant status (TAS), superoxide dismutase (SOD), glutathione peroxidase (GPx), and glutathione reductase (GluRed). Relatively little variation occurred between the two SCC groups below 200 × 10^3^ cells/mL, whereas SCC ≥ 200 × 10^3^ cells/mL was associated with increased pro-inflammatory activity, significant changes in mammary innate-defense proteins, oxidative and metabolic changes, lower body condition, and reduced milk yield. Interleukin-8 showed the strongest independent association with SCC ≥ 200 × 10^3^ cells/mL. Independently estimated breakpoints for 12 inflammatory, innate-defense, and oxidative variables converged within 198–228 × 10^3^ cells/mL. Among the evaluated thresholds, 200 × 10^3^ cells/mL provided 88.0% sensitivity and 82.0% specificity for discrimination of the inflammatory phenotype. These findings identify the region around 200 × 10^3^ cells/mL as a coordinated biological transition rather than a universal diagnostic threshold for mastitis or intramammary infection.

## 1. Introduction

Somatic cell count (SCC) is an established indicator of mammary gland inflammatory status and remains one of the principal parameters used for monitoring udder health in dairy cattle [1,2,3,4]. Its biological interpretation, however, is more complex than its routine diagnostic application implies. An increase in SCC predominantly reflects recruitment of blood-derived leukocytes into the mammary compartment, but the magnitude and cellular composition of this response depend on the nature and intensity of the inflammatory stimulus, stage of lactation, parity, and individual physiological status [1,2,3,4]. SCC should therefore be regarded as an integrated cellular output of mammary immune activation rather than a direct measure of intramammary infection (IMI) or of any single inflammatory pathway. The frequently applied threshold of 200 × 10^3^ cells/mL illustrates this distinction. Its diagnostic utility has been established mainly in relation to the probability of IMI, although sensitivity and specificity vary according to pathogen spectrum, bacteriological definition of infection, sampling strategy, and cow-level characteristics, including parity, stage of lactation, and physiological status [5,6]. Considerably less evidence is available regarding the pathophysiological significance of this region of the SCC distribution. In particular, it remains unresolved whether an SCC approaching 200 × 10^3^ cells/mL merely represents a point of optimal diagnostic discrimination between mammary glands with and without IMI or whether it coincides with a transition in the molecular and cellular processes regulating the mammary inflammatory response.

The increasing use of differential somatic cell count (DSCC) has provided further evidence that total SCC alone incompletely characterizes the mammary immune response. Differences in the functional roles and relative proportions of neutrophils, macrophages, and lymphocytes may reflect changes in mammary immune status that are not fully captured by total SCC [7,8,9]. Recent studies have further shown that leukocyte populations and DSCC are associated with milk characteristics and are influenced by IMI, parity, DIM, and quarter-related factors [10,11,12,13,14,15,16].

At the molecular level, leukocyte recruitment is initiated by recognition of pathogen-associated molecular patterns and endogenous damage-associated signals by mammary epithelial and resident immune cells. Activation of pattern-recognition receptors triggers nuclear factor kappa B (NF-κB)- and mitogen-activated protein kinase (MAPK)-regulated signaling, inducing cytokines, chemokines, adhesion-related mediators, and antimicrobial effectors [17,18]. The magnitude and kinetics of this response are stimulus-dependent, as demonstrated for *Escherichia coli*, *Staphylococcus aureus*, and *Klebsiella pneumoniae* [19,20,21]. Evidence from naturally occurring infections further supports the involvement of these pathways. In cows with naturally occurring *Staphylococcus aureus* subclinical mastitis, alterations in SCC and IL-8-related responses have been reported, while other studies have demonstrated changes in oxidative-status markers in blood and milk, including MDA and total antioxidant capacity, as well as changes in milk proteins and metabolic indices [22,23,24]. Interleukin (IL)-1β, tumor necrosis factor alpha (TNF-α), IL-6, IL-12, interferon gamma (IFN-γ), and IL-17A contribute to inflammatory activation and amplification, whereas IL-10 provides an important counter-regulatory signal limiting excessive pro-inflammatory activity [17,25]. Consequently, SCC represents the downstream cellular manifestation of the balance between inflammatory activation and regulation rather than a uniform inflammatory endpoint. IL-8 is particularly relevant to the relationship between molecular inflammatory activation and SCC because of its central role in neutrophil chemotaxis. Local production of IL-8 promotes recruitment of circulating polymorphonuclear neutrophils across the mammary epithelial barrier, thereby directly linking chemokine signaling to the increase in milk cellularity [17,19]. Puppel et al. [26] demonstrated a progressive increase in milk IL-8 across increasing SCC classes and reported positive relationships between IL-8 and non-esterified fatty acids (NEFA), β-hydroxybutyrate (BHBA), γ-glutamyl transpeptidase (GGT), and aspartate aminotransferase (AST). These associations are mechanistically relevant because they link a mediator of mammary leukocyte recruitment with indices of adipose mobilization, ketogenesis, and hepatic metabolic status. They also raise a broader question: whether the IL-8–SCC relationship represents an isolated chemotactic response or forms part of a coordinated immunometabolic transition accompanying increasing mammary cellularity.

Mammary innate defense extends beyond cytokine-dependent leukocyte recruitment. Lactoferrin, lysozyme, and lactoperoxidase constitute complementary antimicrobial systems with distinct molecular mechanisms. Lactoferrin limits microbial proliferation through high-affinity iron binding and additionally exhibits direct antimicrobial and immunomodulatory activities; lysozyme hydrolyses β-1,4 glycosidic linkages within bacterial peptidoglycan; and the lactoperoxidase system catalyzes oxidation of thiocyanate in the presence of hydrogen peroxide, generating antimicrobial hypothiocyanite [17,27]. Changes in these proteins may therefore indicate modification of the local antimicrobial environment independently of changes in cytokine concentrations.

The relationship between lactoferrin and mammary inflammatory status is particularly informative in the context of systemic metabolism. Konečný et al. [28] demonstrated associations among milk lactoferrin, SCC, metabolic profiles, and milk composition, while Puppel et al. [29] reported relationships between IL-8 and metabolic indices. Together, these findings indicate that distinct components of mammary defense may be linked to the systemic metabolic state of the cow. Proteomic studies further demonstrate that mammary inflammation and increased milk cellularity are accompanied by extensive changes in proteins and pathways related to innate immunity, antimicrobial defense, cellular stress, and leukocyte function [29,30], supporting the evaluation of multiple functionally distinct biomarkers across the SCC continuum. A second mechanistic axis involves the interaction between inflammation and redox regulation. Neutrophil activation generates reactive oxygen species (ROS) through the NADPH oxidase system; although essential for antimicrobial defense, excessive ROS production may overwhelm antioxidant capacity and promote oxidative damage [31,32]. This relationship is bidirectional, as ROS can modulate inflammatory signaling, while inflammatory cell recruitment further increases ROS-generating capacity; oxidized lipids and lipid-derived mediators may additionally contribute to both amplification and resolution of inflammation [33]. In dairy cows, the oxidative and metabolic demands of lactation may further limit the capacity to maintain redox homeostasis during inflammatory challenge [34]. Inflammatory–redox interactions are also closely linked to systemic energy metabolism. Increased adipose tissue mobilization elevates circulating NEFA, while hepatic oxidation of mobilized fatty acids contributes to BHBA production; these metabolic changes may in turn influence leukocyte function, inflammatory responsiveness, and oxidative metabolism [35,36,37]. Conversely, immune activation alters nutrient requirements and partitioning, indicating a reciprocal relationship between metabolic adaptation and inflammatory activity.

Metabolomic evidence has reinforced this concept by identifying alterations in lipid, amino-acid, and energy metabolic pathways in association with bovine mammary inflammation [38]. Within this framework, the observations of Puppel et al. [26] and Konečný et al. [28] are complementary. The former linked IL-8 and SCC with indices of lipid mobilization, ketogenesis, and hepatic status, whereas the latter linked lactoferrin and SCC with metabolic profiles and milk composition. These studies provide evidence that both inflammatory signaling and innate mammary defense are associated with systemic metabolic characteristics. Whether these relationships converge at a common stage of increasing SCC, however, has not been established.

Body condition score (BCS) may further modify this interaction because BCS integrates longer-term differences in energy reserves and their mobilization. Its relationship with inflammatory status cannot therefore be interpreted independently of circulating NEFA and BHBA or of oxidative status. At the same time, inflammatory activation may influence nutrient allocation away from milk synthesis toward immune and repair processes [35,36,37]. This provides a physiological basis for the established negative association between SCC and milk yield [39,40]. The recent demonstration of quarter-level relationships among SCC, DSCC, milk yield, and milk composition by Fonseca et al. [15] further indicates that changes in mammary cellularity have functional consequences that depend on the underlying inflammatory state.

Despite extensive evidence linking SCC with mammary inflammation, oxidative stress, and metabolic status, these biological domains have generally been examined separately or in relation to predefined SCC categories. Consequently, it remains unclear whether increasing SCC is associated with gradual and independent changes in individual biomarkers or whether multiple components of the inflammatory, innate-defense, oxidative, and metabolic response converge within a common region of the SCC continuum. To our knowledge, the convergence of independently estimated change points across inflammatory, mammary innate-defense, and oxidative responses within the continuous SCC distribution has not previously been evaluated in a single integrated study. This distinction is particularly relevant around 200 × 10^3^ cells/mL, a threshold widely used in udder-health assessment but whose broader biological significance remains incompletely defined. Therefore, the aim of the present study was to characterize inflammatory, mammary innate-defense, oxidative, metabolic, and production-related changes across increasing SCC in clinically healthy Holstein–Friesian cows without detectable intramammary infection and to determine whether these responses converge within a common SCC range. In addition to comparisons across predefined SCC categories, continuous breakpoint analysis was used to estimate independently the SCC values at which individual biological markers changed their response pattern. We hypothesized that the region around 200 × 10^3^ cells/mL would correspond to a coordinated biological transition characterized by increased pro-inflammatory activity, altered mammary innate-defense responses, deterioration of oxidative balance, and concomitant metabolic and production-related changes.

## 2. Results

Milk yield was similar in cows with SCC < 100 and 100–199 × 10^3^ cells/mL (34.1 and 33.6 kg/day, respectively) but decreased to 31.7 kg/day in cows with SCC 200–399 × 10^3^ cells/mL and further to 28.9 kg/day in cows with SCC ≥ 400 × 10^3^ cells/mL (*p* < 0.001) (Table 1). A similar pattern was observed for milk fat concentration (*p* < 0.001). Protein concentration did not differ significantly among SCC categories (*p* = 0.067), whereas CLA c9,t11 concentration was lower in the highest SCC category than in the <100 × 10^3^ cells/mL group (*p* = 0.006).

Inflammatory cytokine concentrations differed markedly across SCC categories (Table 2). Concentrations of IL-1β, IL-6, IL-8, TNF-α, IL-12, and IL-17A increased at SCC ≥ 200 ×10^3^ cells/mL, whereas IL-10 decreased. All overall associations remained significant after correction for multiple testing (all FDR-adjusted *q* < 0.001). The most pronounced increase was observed for IL-8, which rose from 0.69 ± 0.31 and 0.78 ± 0.39 ng/mL in the two lowest SCC categories to 2.42 ± 1.51 and 7.68 ± 5.20 ng/mL in the 200–399 and ≥400 ×10^3^ cells/mL groups, respectively. In contrast, IL-10 concentrations decreased at SCC ≥ 200 ×10^3^ cells/mL and were lowest in the ≥400 ×10^3^ cells/mL group (*p* < 0.001).

The balance between pro- and anti-inflammatory cytokines shifted toward a pro-inflammatory profile with increasing SCC, with the most evident separation occurring at SCC ≥ 200 × 10^3^ cells/mL (Table 3). The IL-6/IL-10, TNF-α/IL-10, IL-1β/IL-10, and IL-17A/IL-10 ratios remained similar in cows with SCC < 100 and 100–199 × 10^3^ cells/mL, but increased markedly in the 200–399 × 10^3^ cells/mL category and increased further at SCC ≥ 400 × 10^3^ cells/mL. Consistently, PCI shifted from negative values below 200 × 10^3^ cells/mL to positive values in the two higher-SCC categories. All overall associations remained significant after correction for multiple testing (all FDR-adjusted *q* < 0.001).

Milk bioactive proteins showed consistent changes with increasing SCC (Table 4). Lactoferrin, lysozyme, lactoperoxidase, and β-lactoglobulin concentrations were similar in the two lower-SCC categories, increased at SCC 200–399 × 10^3^ cells/mL, and reached their highest values at SCC ≥ 400 × 10^3^ cells/mL. The overall SCC-category effect remained significant for all four proteins after correction for multiple testing (all FDR-adjusted *q* < 0.001).

Metabolic and hepatic biomarkers also varied across SCC categories (Table 5). NEFA and BHBA concentrations increased with increasing SCC, whereas glucose concentrations decreased. AST and GGT activities showed a parallel increase across the higher-SCC categories. Differences were most evident from SCC ≥ 200 × 10^3^ cells/mL onward, while values in the <100 and 100–199 × 10^3^ cells/mL categories remained comparatively similar. All overall SCC-category associations remained significant after correction for multiple testing (all FDR-adjusted *q* < 0.001).

Oxidative status differed across SCC categories (Table 6). MDA concentrations increased markedly at SCC ≥ 200 × 10^3^ cells/mL, whereas TAS decreased, indicating a shift toward greater oxidative pressure and reduced overall antioxidant capacity. SOD activity was also higher in the two highest SCC categories, while GPx activity remained unchanged. Glutathione reductase showed a smaller but significant overall increase across SCC categories. After correction for multiple testing, the associations remained significant for MDA, TAS, SOD, and GluRed, whereas GPx remained non-significant.

Planned contrasts between adjacent SCC categories revealed little evidence of biomarker separation between the two lowest SCC groups (<100 and 100–199 × 10^3^ cells/mL; Table 7). In contrast, the transition from 100–199 to 200–399 × 10^3^ cells/mL was accompanied by coordinated changes across inflammatory, innate-defense, oxidative, and metabolic markers. These associations were retained after FDR correction. Further changes were observed between 200–399 and ≥400 × 10^3^ cells/mL, indicating continued progression of the biological response at higher SCC. Thus, the categorical analysis identified the most extensive shift between the 100–199 and 200–399 × 10^3^ cells/mL categories, consistent with—but analytically independent of—the transition region identified by continuous segmented regression.

Spearman rank correlation analysis showed consistent monotonic associations between SCC and inflammatory, innate-defense, metabolic, oxidative, and production-related markers (Table 8). The strongest positive associations were observed for IL-8 (ρ = 0.77) and PCI (ρ = 0.74), followed by IL-6, TNF-α, IL-1β, IL-17A, lysozyme, and lactoperoxidase. SCC was also positively associated with NEFA, BHBA, AST, GGT, and MDA, whereas inverse associations were observed for IL-10, TAS, BCS, and milk yield. All correlations remained significant after FDR correction (all *q* < 0.001).

In the multivariable logistic regression analysis, inflammatory, metabolic, and oxidative stress markers remained independently associated with SCC ≥ 200 × 10^3^ cells/mL in the final adjusted model (Table 9). Among the inflammatory markers, IL-8 showed the strongest positive association. Each 1 ng/mL increase in IL-8 was associated with an 84% increase in the adjusted odds of SCC ≥ 200 × 10^3^ cells/mL (OR = 1.84, 95% CI: 1.60–2.13; *p* < 0.001). Positive associations were also observed for IL-6 (OR = 1.31 per 10 pg/mL, 95% CI: 1.16–1.49; *p* < 0.001) and IL-17A (OR = 1.28 per 10 pg/mL, 95% CI: 1.09–1.51; *p* = 0.003). In contrast, IL-10 was inversely associated with the outcome (OR = 0.81 per 10 pg/mL, 95% CI: 0.69–0.95; *p* = 0.010). Independent associations were also evident for markers outside the cytokine profile. A 0.5 mmol/L increase in NEFA was associated with higher odds of SCC ≥ 200 × 10^3^ cells/mL (OR = 1.42, 95% CI: 1.11–1.82; *p* = 0.006), as was a 10 µmol/L increase in MDA (OR = 1.29, 95% CI: 1.07–1.56; *p* = 0.008). Higher BCS was associated with lower odds of the outcome (OR = 0.71 per 1-point increase, 95% CI: 0.53–0.95; *p* = 0.021). DIM, lactation number, and milk yield were not independently associated with SCC ≥ 200 × 10^3^ cells/mL in the adjusted model.

Segmented regression identified significant changes in the SCC–biomarker relationships, with independently estimated change points clustering within a narrow SCC interval (Table 10). Across the 12 inflammatory, innate-defense, and oxidative markers, breakpoint estimates ranged from 198 to 228 × 10^3^ cells/mL. Because SCC was analyzed continuously and no breakpoint location was specified a priori, this convergence was independent of the predefined SCC categories. Comparable estimates were obtained for IL-6 (207 × 10^3^ cells/mL), lactoperoxidase (211 × 10^3^ cells/mL), TAS (214 × 10^3^ cells/mL), IL-1β (216 × 10^3^ cells/mL), lysozyme (218 × 10^3^ cells/mL), IL-10 (219 × 10^3^ cells/mL), TNF-α (221 × 10^3^ cells/mL), MDA (226 × 10^3^ cells/mL), and IL-12 (228 × 10^3^ cells/mL). Thus, despite representing different components of the inflammatory, innate-defense, and oxidative response, all estimated breakpoints were concentrated within a 30 × 10^3^ cells/mL interval.

Among the evaluated SCC cut-offs, ≥200 × 10^3^ cells/mL provided the best balance between sensitivity and specificity for identifying the inflammatory phenotype (Table 11). At this threshold, sensitivity was 88.0% and specificity was 82.0%, with a PPV of 68.9% and an NPV of 93.4%. The corresponding Youden index was 0.70, compared with 0.48, 0.61, and 0.51 for thresholds of ≥100, ≥300, and ≥400 × 10^3^ cells/mL, respectively. The ≥100 × 10^3^ cells/mL threshold provided the highest sensitivity (94.0%) but substantially lower specificity (54.0%), whereas the ≥400 × 10^3^ cells/mL threshold provided the highest specificity (93.0%) but the lowest sensitivity (58.0%).

ROC analysis showed varying degrees of correspondence between the evaluated inflammatory measures and the study-derived inflammatory phenotype (Table 12). PCI showed the highest AUC (0.951; 95% CI: 0.930–0.968), with sensitivity of 90.0% and specificity of 88.2%. Among individual cytokines, IL-8 showed the highest AUC (0.934; 95% CI: 0.910–0.954), with sensitivity of 88.8% and specificity of 86.5%. IL-6, IL-17A, TNF-α, and IL-1β also showed high correspondence with the inflammatory phenotype, whereas lower AUC values were observed for IL-12 and IL-10. Because the phenotype was derived from the same cytokine panel, these ROC estimates represent internal correspondence with the study-derived phenotype rather than independent diagnostic validation.

Integrated biomarker models showed high correspondence with the study-derived inflammatory phenotype, which was largely retained after internal bootstrap validation (Table 13). IL-8 alone yielded an apparent AUC of 0.934 and an optimism-corrected AUC of 0.930. Addition of IL-6 and TNF-α increased the corrected AUC to 0.943, whereas further inclusion of IL-17A and IL-10 resulted in a corrected AUC of 0.951. PCI alone showed similarly high discrimination (apparent AUC = 0.951; optimism-corrected AUC = 0.945). Integration of PCI with MDA and TAS produced the highest corrected discrimination (AUC = 0.954; 95% CI: 0.932–0.970). Although the fully integrated model containing PCI, MDA, TAS, NEFA, and BCS yielded the highest apparent AUC (0.967), its greater bootstrap optimism resulted in the same corrected AUC of 0.954 (95% CI: 0.931–0.971). Thus, discrimination remained high after correction for within-sample optimism, while the incremental gain associated with increasing model complexity was attenuated after internal validation.

Direct comparison of cows with SCC < 200 and ≥200 × 10^3^ cells/mL revealed pronounced differences across inflammatory, oxidative, metabolic, and production-related parameters (Table 14). The largest relative change was observed for IL-8, which increased from 0.72 ± 0.34 to 4.72 ± 4.46 ng/mL (+556.7%; Cohen’s d = 1.70; *p* < 0.001). Marked increases were also observed for IL-17A (+128.2%; d = 1.70), IL-1β (+110.3%; d = 1.65), IL-6 (+108.6%; d = 1.57), and TNF-α (+108.1%; d = 1.58), whereas the anti-inflammatory cytokine IL-10 decreased by 19.1% (d = −0.47; *p* < 0.001). Differences were also evident in oxidative and metabolic status. MDA was 26.2% higher (d = 0.90), whereas TAS was 29.8% lower (d = −0.48) in cows with SCC ≥ 200 × 10^3^ cells/mL. NEFA increased by 64.1% (d = 0.70). These changes were accompanied by a 9.5% reduction in BCS (d = −0.68) and a 10.2% reduction in milk yield (d = −0.44). All evaluated differences were statistically significant (*p* < 0.001). Collectively, these findings show that cows with SCC ≥ 200 × 10^3^ cells/mL differed markedly from those with SCC < 200 × 10^3^ cells/mL, particularly in their inflammatory profile, with additional moderate differences in oxidative and metabolic status, body condition, and milk production.

## 3. Discussion

An important feature of the present study was that mammary health status was assessed at the quarter level and independently of SCC. Cows were not classified as free of detectable IMI simply because they had low SCC; instead, milk from individual quarters underwent microbiological examination, and cows with microbiological evidence of infection in any quarter were excluded. Consequently, the inflammatory transition identified along the SCC continuum cannot be attributed simply to an SCC-based definition of mammary health. The present study provides evidence that the relationship between SCC and mammary inflammatory status is not adequately described by a simple linear increase across the range of cell counts. Rather, the data indicate a relatively narrow region in which several components of the inflammatory response changed concurrently. The clearest separation occurred between cows with SCC of 100–199 and 200–399 × 10^3^ cells/mL, while comparatively little differentiation was evident between the two groups below 200 × 10^3^ cells/mL. Importantly, the same pattern was recovered when SCC was treated as a continuous variable: independently estimated breakpoints for inflammatory, innate-defense, and oxidative markers were confined to 198–228 × 10^3^ cells/mL. The proximity of these estimates, obtained for biomarkers representing different biological processes, is central to the interpretation of the study. It suggests that the region around 200 × 10^3^ cells/mL corresponded to a coordinated change in mammary inflammatory status in the cows examined, rather than merely reflecting the conventional categorization of SCC. This interpretation requires a clear distinction between inflammation and intramammary infection. The two are related, but they are not interchangeable. SCC is an indicator of the cellular response within the mammary gland and is consequently strongly associated with intramammary infection, yet neither a low SCC excludes infection nor an elevated SCC establishes its presence. This limitation has been recognized since the early work of Dohoo and Leslie [1] and Schepers et al. [2] and remains relevant despite considerable advances in mastitis diagnostics. Schukken et al. [3] emphasized the probabilistic nature of SCC-based classification, while Schwarz et al. [4] demonstrated substantial variation in milk leukocyte populations according to mammary health status. More recent work has further shown that interpretation of total SCC is improved when information on leukocyte composition is considered [7,8,9,37]. This issue has become particularly apparent with the introduction of differential somatic cell count. Fonseca et al. [11] recently demonstrated at quarter level that IMI was the principal source of variation in DSCC, but parity, DIM, and season also contributed to its variability. Their companion study showed that healthy and subclinically affected quarters differed in DSCC distributions and that interpretation of the cellular response depended on total SCC as well as leukocyte composition [13]. Similarly, Mondini et al. [14] found that parity and stage of lactation influenced both SCC and the relative proportions of neutrophils, lymphocytes, and macrophages in milk. Taken together, these observations argue against treating any single SCC value as an invariant boundary between physiological and pathological states. The present findings do not contradict this literature. They address a different question. The cows included here were clinically healthy and free of the mastitis, metabolic disorders, and locomotor disease specified by the exclusion criteria; intramammary infection was additionally excluded using the diagnostic procedures described in the Materials and Methods. Consequently, the breakpoint identified in this study should not be interpreted as an estimate of the SCC that best detects IMI. What emerged at approximately 200 × 10^3^ cells/mL was a change in the host response in a deliberately selected population without evidence of overt mammary disease. This distinction is fundamental because it shifts the interpretation of SCC from pathogen detection toward the biological state of the mammary gland.

The cytokine profile provides the most direct evidence for such a shift. Concentrations of IL-1β, IL-6, IL-8, TNF-α, IL-12, and IL-17A increased once SCC entered the higher ranges, whereas IL-10 changed in the opposite direction. These mediators do not represent a single inflammatory pathway. IL-1β and TNF-α participate in the initiation and amplification of innate inflammatory signaling; IL-6 has both local and systemic actions; IL-8 is closely involved in neutrophil recruitment; and IL-17 contributes to neutrophilic immunity and amplification of local inflammatory responses. The concurrent behavior of these mediators is therefore more informative than an isolated increase in any one cytokine. This pattern is consistent with the established biology of the bovine mammary immune response. Riollet et al. [19] showed that mammary inflammation is accompanied by coordinated changes in cytokines involved in leukocyte recruitment and activation, while Alluwaimi [25] subsequently emphasized that cytokine interactions, rather than individual cytokine concentrations, determine the character of the mammary immune response. These mechanisms have since been confirmed and refined in experimental models of pathogen-specific mastitis, which show that the magnitude and kinetics of cytokine responses vary substantially with the initiating stimulus [18,20,21]. Thus, the cytokine pattern observed here is compatible with low-grade activation of mammary immune mechanisms, but should not be equated with the much stronger response occurring during experimentally induced or naturally occurring mastitis.

IL-8 was exceptional in both the magnitude and consistency of its association with SCC. It showed the strongest monotonic association with SCC, the greatest discriminatory capacity among individual cytokines, and the largest adjusted odds ratio in the multivariable analysis. Its estimated breakpoint, 198 × 10^3^ cells/mL, was also at the lower boundary of the breakpoint range. These findings are biologically coherent with the function of IL-8 as a major chemoattractant for bovine neutrophils. Recruitment of polymorphonuclear cells from the circulation is one of the earliest and quantitatively most important components of the mammary inflammatory response; consequently, a close relationship between IL-8 and the cellular component measured as SCC is expected [17,19]. The result also extends our previous observations. Puppel et al. [26] reported a progressive increase in IL-8 across SCC classes and demonstrated associations between IL-8, SCC, and indices of metabolic status. The present data reproduce the strong SCC–IL-8 relationship, but add two pieces of information that were not available from the earlier categorical comparison: IL-8 remained independently associated with SCC ≥ 200 × 10^3^ cells/mL after adjustment for potential covariates, and piecewise analysis located the change in its relationship with SCC at approximately 198 × 10^3^ cells/mL. Thus, the current results support IL-8 not simply as another biomarker associated with SCC, but as one of the components most closely linked to the transition identified in the present population. IL-10 provides an important counterpoint to this pro-inflammatory response. Its decline with increasing SCC resulted in pronounced increases in IL-1β/IL-10, IL-6/IL-10, TNF-α/IL-10, and IL-17A/IL-10. The direction of these ratios is important because the inflammatory state of the mammary gland depends on the balance between activating and regulatory signals. IL-10 limits excessive inflammatory activity through suppression of several macrophage- and lymphocyte-mediated pathways, and its relative abundance can therefore modify the biological consequences of otherwise comparable pro-inflammatory signaling [17,25]. In the present study, the transition above approximately 200 × 10^3^ cells/mL was consequently characterized not only by higher concentrations of pro-inflammatory cytokines, but also by a shift in the balance between pro- and anti-inflammatory signaling. The behavior of the Pro-inflammatory Cytokine Index should be considered in this context. PCI showed greater correspondence with the study-defined inflammatory phenotype than any single cytokine, with an AUC of 0.951. This result is statistically plausible because the index integrates information from several correlated mediators, but it is also biologically informative: no single cytokine accounted for the entire inflammatory pattern. PCI should not, however, be presented as a validated diagnostic biomarker. It was developed and evaluated in the same study population and therefore requires external validation before its performance can be generalized.

Changes in lactoferrin, lysozyme, and lactoperoxidase indicate that the response associated with increasing SCC extended beyond cytokine signaling to soluble components of mammary innate defense. Lysozyme and lactoperoxidase contribute to antimicrobial defense in milk through mechanisms distinct from cytokine-mediated leukocyte recruitment [17]. Importantly, their estimated breakpoints occurred within the same transition region as those of the inflammatory mediators. Although lactoferrin also increased with SCC, its concentration may be influenced by mammary epithelial secretion, stage of lactation, and milk production [27]; therefore, its increase should be interpreted as part of the broader mammary response rather than as a specific indicator of intramammary infection. A second major feature of the higher-SCC phenotype was deterioration of oxidative balance. MDA increased while TAS declined, whereas SOD activity increased and GPx remained comparatively stable. Considered in isolation, greater SOD activity might appear inconsistent with oxidative stress; however, together with higher MDA and lower TAS, it is more consistent with a compensatory enzymatic response that did not fully preserve overall redox balance. This interpretation agrees with the established biological coupling between inflammation and oxidative stress in dairy cattle. Activated phagocytes generate reactive oxygen species during the respiratory burst, while excessive ROS production relative to antioxidant capacity promotes lipid peroxidation and oxidative damage [31,32,34]. The proximity of the oxidative-marker breakpoints to those of the inflammatory and innate-defense markers further indicates that deterioration of redox balance emerged within the same SCC region. Because the data are cross-sectional, however, the temporal direction of these relationships cannot be resolved. The metabolic findings broaden this phenotype beyond the mammary gland. Increasing SCC was accompanied by higher NEFA and BHBA and lower BCS, while AST and GGT were also higher in the upper SCC categories. In the adjusted model, NEFA remained positively associated with SCC ≥ 200 × 10^3^ cells/mL and BCS retained an inverse association. These findings are compatible with the well-established interaction between metabolic adaptation and immune function in dairy cows. Increased adipose mobilization elevates circulating NEFA, modifies hepatic metabolism, and can influence leukocyte and mammary epithelial function [35,36,37]. The present design does not establish metabolic stress as a cause of the mammary inflammatory response: inflammatory signaling can itself alter feed intake, nutrient partitioning, and lipid metabolism, making bidirectional relationships plausible. The complementary behavior of NEFA and BCS is nevertheless informative. NEFA reflects relatively acute adipose mobilization, whereas BCS represents accumulated energy reserves; their opposite adjusted associations indicate that the higher-SCC phenotype coincided with both current metabolic mobilization and lower energy reserves. Because DIM, parity, and milk yield did not remain independently associated in the adjusted model, these metabolic differences are unlikely to be explained solely by obvious variation in lactation stage or production. Temporal sequence, however, cannot be inferred from these data.

Milk yield showed another relevant pattern. Production differed little between the two SCC categories below 200 × 10^3^ cells/mL and declined thereafter. The association between increasing SCC and milk loss is one of the most reproducible observations in dairy epidemiology [39,40], but recent work indicates that the relationship is more complex than total SCC alone suggests. Fonseca et al. [15], analysing quarter-level milk production jointly with SCS and DSCC, showed that combining information on total and differential cell counts provides additional insight into production changes associated with mammary inflammation. In our analysis, milk yield was no longer independently associated with SCC ≥ 200 × 10^3^ cells/mL after adjustment. The reduction in production is therefore better regarded as part of the phenotype accompanying the inflammatory transition than as an independent explanation for it.

A central methodological question is whether the apparent transition around 200 × 10^3^ cells/mL reflects a biological feature of the data or merely the predefined categorization of SCC. The strongest evidence against a purely categorical artifact comes from the segmented regression, in which SCC was treated continuously and no breakpoint location was imposed a priori. Despite this, independently estimated breakpoints for 12 inflammatory, innate-defense, and oxidative biomarkers clustered within a narrow interval of 198–228 × 10^3^ cells/mL. This convergence is more informative than any single breakpoint because the markers represent distinct biological processes and were estimated independently. The categorical results were consistent with the same pattern: comparatively little separation was observed between the two groups below 200 × 10^3^ cells/mL, whereas differences became more evident between 100–199 and 200–399 × 10^3^ cells/mL. In addition, among the prespecified candidate thresholds, 200 × 10^3^ cells/mL provided the most favorable balance of sensitivity and specificity for classifying an inflammatory phenotype defined without SCC. These analyses should therefore be viewed as complementary: the continuous models locate a transition region, while the categorical and threshold analyses describe its biological and classificatory expression.

The latter analysis requires particular caution. Sensitivity and specificity in this context describe classification of the study-defined inflammatory phenotype, not diagnosis of mastitis or IMI. Direct comparison with microbiological studies would therefore be inappropriate. Published estimates of the optimal SCC threshold for IMI vary considerably because they depend on sample type, pathogen definition, parity, DIM, prevalence, and the diagnostic reference standard [3,5,6]. The current DSCC literature reinforces this point. Fonseca et al. [11,13] found that IMI, DIM, parity, and pathogen group influenced cellular characteristics of milk, while Mondini et al. [14] showed substantial physiological variation in leukocyte composition even outside overt mastitis. Accordingly, the present estimate should not be promoted as a new universal mastitis threshold.

The contribution of the study therefore lies primarily in the convergence of evidence rather than in any single numerical cut-off. A similar SCC region emerged from conventional group comparisons, continuous breakpoint modelling, and classification against a phenotype defined independently of SCC. More importantly, the changes contributing to this convergence were distributed across several biological domains, including pro- and anti-inflammatory cytokine signaling, selected innate-defense proteins, oxidative balance, and metabolic status. The close clustering of independently estimated breakpoints across these systems supports the interpretation of approximately 200 × 10^3^ cells/mL as a biologically informative transition region in this population, while not establishing it as an invariant diagnostic boundary.

The integrated biomarker models further support the multidimensional nature of the biological changes associated with increasing SCC, although their incremental performance should be interpreted cautiously. PCI alone showed high correspondence with the study-derived inflammatory phenotype. Addition of the oxidative markers MDA and TAS produced a modest but statistically significant increase in apparent discrimination (ΔAUC = 0.012, 95% CI: 0.003–0.021; *p* = 0.009), indicating that oxidative status provided information beyond that captured by the inflammatory cytokine profile alone. In contrast, further inclusion of NEFA and BCS resulted in only a small and non-significant increase in apparent AUC (ΔAUC = 0.004, 95% CI: −0.002–0.010; *p* = 0.184). This distinction was reinforced by internal bootstrap validation: the inflammatory–oxidative model and the fully integrated inflammatory–oxidative–metabolic model both yielded an optimism-corrected AUC of 0.954, despite the higher apparent AUC of the latter model. Thus, increasing model complexity beyond the inflammatory–oxidative domain did not materially improve internally validated discrimination. Nevertheless, the associations of NEFA and BCS with the higher-SCC state remain biologically relevant, as they indicate that the transition in mammary inflammatory activity occurred in parallel with changes in systemic metabolic status and body condition.

Several aspects of the study limit the extent to which these findings can be generalized. First, all cows originated from a single herd and represented a deliberately restricted range of parity and days in milk. Although this design reduced variation attributable to established physiological determinants of SCC, it also limits external validity. Previous studies have shown that parity and stage of lactation influence both total SCC and leukocyte composition in milk [11,14]. Validation in independent herds differing in production level, management, lactation structure, and pathogen pressure is therefore required to determine whether the breakpoint region identified here is reproducible across populations. Second, the inflammatory phenotype used in the classification analyses was derived empirically from the cytokine distributions observed in the present population. Although it was defined independently of SCC and PCI, its component thresholds were study-specific, and the resulting ROC estimates should therefore be interpreted as measures of internal correspondence rather than externally validated diagnostic performance. This consideration is particularly relevant for analyses involving individual cytokines and PCI, because the same cytokine panel contributed to construction of the reference phenotype. Independent prospective validation using a predefined phenotype would be required to establish the reproducibility and predictive value of these associations. Third, the observational design precludes inference about the temporal or causal relationships among mammary inflammation, oxidative imbalance, and metabolic status. The present data cannot determine whether changes in NEFA, BCS, and oxidative markers precede the increase in mammary inflammatory activity, develop concurrently with it, or arise partly as a consequence of the inflammatory response. Longitudinal within-cow sampling across the SCC continuum would be particularly informative for establishing the temporal sequence of changes in IL-8, SCC, oxidative markers, and metabolic indicators. Within these constraints, the present findings support an interpretation of SCC as a continuum reflecting changes in mammary biological status rather than as a simple binary separator between healthy and mastitic states. In clinically healthy cows without detectable intramammary infection, the region around 200 × 10^3^ cells/mL coincided with coordinated changes in pro- and anti-inflammatory cytokine activity, selected components of mammary innate defense, oxidative balance, and systemic metabolic status. Particularly important was the close clustering of independently estimated breakpoints across biomarkers representing distinct biological domains. Because these change points were derived from continuous SCC rather than imposed by the predefined SCC categories, their convergence argues against the observed transition being solely an artefact of categorical classification. The accompanying reduction in milk yield and body condition further indicates that the higher-SCC state was associated with biologically meaningful changes extending beyond local inflammatory signaling. Nevertheless, the present data do not establish 200 × 10^3^ cells/mL as a diagnostic cut-off for mastitis or intramammary infection. Rather, they identify the region around this value as a candidate biological transition zone in the population examined, the reproducibility and broader relevance of which require independent prospective validation.

## 4. Materials and Methods

### 4.1. Animals, Housing, and Experimental Design

The study included 580 Polish Holstein–Friesian dairy cows, representing the predominant dairy cattle population used for milk production in Poland, in their second or third lactation. The animals were maintained at the experimental dairy farm of the Warsaw University of Life Sciences (WULS-SGGW), located in Obory (101 Obory, 05-520 Konstancin-Jeziorna, Masovian Voivodeship, Poland). Data were collected over a two-year period, and cows were evaluated between 45 and 65 days in milk (DIM). The distribution and general characteristics of cows across SCC categories are presented in Table 15. Of the 580 cows included in the study, 49.1% had SCC < 100 × 10^3^ cells/mL, 23.3% had SCC of 100–199 × 10^3^ cells/mL, 15.5% had SCC of 200–399 × 10^3^ cells/mL, and 12.1% had SCC ≥ 400 × 10^3^ cells/mL. Body condition score differed significantly among SCC categories (*p* < 0.001), remaining similar in the two lowest SCC groups but decreasing at SCC ≥ 200 × 10^3^ cells/mL, with the lowest BCS observed in cows with SCC ≥ 400 × 10^3^ cells/mL. Body weight also differed among groups (*p* = 0.008), with cows in the highest SCC category having lower body weight than those in the lowest categories. In contrast, DIM did not differ among SCC categories (*p* = 0.631). Milk yield followed the previously observed pattern, remaining similar below 200 × 10^3^ cells/mL and decreasing significantly in the two higher SCC categories (*p* < 0.001).

All cows received the same total mixed ration (TMR) throughout the study period from March to October (Table 16). The ration was formulated according to the INRA feeding system and was offered ad libitum. Total dry matter supplied was 21.20 kg/day, with a reported daily dry matter intake of 19.90 kg/day and a net energy for lactation concentration of 1.75 Mcal/kg. The TMR was based predominantly on maize silage and alfalfa silage, supplemented with ensiled maize grain, soybean meal, rapeseed meal, and mineral components. The unchanged TMR formulation throughout the study period provided consistent nutritional conditions for the evaluated animals.

Cows were milked twice daily in a herringbone milking parlour. Milk and blood samples were collected on the same sampling day, before the morning feeding.

Individual cow-level composite milk samples (approximately 250 mL), representing milk from all four udder quarters, were obtained during the morning milking and collected into sterile plastic containers containing Mlekostat CC preservative (Polmass, Bydgoszcz, Poland). Following collection, the samples were kept refrigerated during transport to the laboratory of the Warsaw University of Life Sciences. In the laboratory, milk was divided into aliquots according to the requirements of the individual analytical procedures. The samples were subsequently used for determination of milk composition, somatic cell count (SCC), inflammatory biomarkers, whey protein concentrations, and fatty acid composition. On the same day, blood samples (10 mL) were collected from the jugular vein into sterile Vacuette^®^ tubes (Greiner Bio-One, Kremsmünster, Austria) before morning feeding. The samples were transported to the laboratory under controlled conditions and centrifuged at 1800× *g* for 15 min at 4 °C. Serum was separated after centrifugation and stored at −20 °C until further analysis. The serum samples were used for the determination of metabolic indicators, liver-associated enzymes, and markers of oxidative status. The same sampling and sample-handling procedures were applied throughout the study period. Milk and blood samples were collected from all 580 cows included in the study. The numbers of cows/samples in the SCC categories were 285, 135, 90, and 70 for SCC < 100, 100–199, 200–399, and ≥400 × 10^3^ cells/mL, respectively. Milk was used for determination of milk composition, SCC, inflammatory cytokines, mammary innate-defense proteins, and fatty acid composition, whereas serum was used for metabolic, hepatic, and oxidative-status analyses. No analyte was measured in both matrices. This matrix-specific analytical approach was intended to provide complementary information on local mammary responses from milk and systemic metabolic and oxidative status from serum; parallel assessment of the same analytes in both matrices was beyond the scope of the present study.

Body condition was evaluated in each cow by a single trained assessor according to the five-point BCS scale of Edmonson et al. [41]. Scores were assigned in 0.25-point intervals. The assessment combined visual inspection with palpation of the principal anatomical landmarks used to estimate subcutaneous fat deposition, particularly the lumbar and pelvic regions, including the hooks, pins, rump, and tail-head area. The same evaluator performed all assessments throughout the study to limit inter-observer variation.

All cows included in the study were clinically healthy and remained under regular veterinary supervision throughout the study period. Health status was assessed using herd health records, routine veterinary examinations, and clinical evaluation performed at the time of sampling. Cows with metabolic disorders, hoof lesions or locomotor problems, systemic disease, or any other condition requiring veterinary treatment were excluded. Particular attention was given to mammary gland health. None of the included cows showed clinical signs of mastitis. Udder health was evaluated at the quarter level: milk samples were collected separately from each mammary quarter and subjected to comprehensive microbiological examination, supplemented by molecular testing by PCR. Cows with microbiological or molecular evidence of intramammary infection in any quarter were excluded. Importantly, SCC itself was not used as an exclusion criterion for IMI, allowing clinically healthy cows across the full SCC range investigated in the study to be retained provided that quarter-level examination showed no detectable evidence of infection. Thus, the SCC categories represented differences in milk somatic cell concentration among clinically healthy cows without detectable IMI at the time of sampling rather than groups defined by the presence or severity of mastitis. Accordingly, the SCC ≥ 400 × 10^3^ cells/mL category represented the highest analytical SCC stratum and was not considered a diagnostic category of clinical or subclinical mastitis. Only animals meeting all predefined health criteria were retained for further analyses.

### 4.2. Determination of Metabolic Biomarkers

Serum non-esterified fatty acids (NEFA), β-hydroxybutyrate (BHBA), aspartate aminotransferase (AST), glucose and γ-glutamyl transferase (GGT) were determined to characterize the metabolic and hepatic status of the cows. All measurements were performed using a BS-800M automated biochemical analyzer (PZ Cormay S.A., Warsaw, Poland). NEFA and BHBA were expressed as serum concentrations, whereas AST and GGT were reported as enzyme activities.

### 4.3. Determination of Oxidative Stress Biomarkers

Oxidative status was assessed in serum by determination of malondialdehyde (MDA) and total antioxidant status (TAS), as well as the activities of glutathione reductase (GluRed), glutathione peroxidase (GPx), and superoxide dismutase (SOD).

Malondialdehyde was determined by the thiobarbituric acid reactive substances (TBARS) method. For the assay, 250 μL of serum was mixed with 25 μL of 0.2% 2,6-bis(1,1-dimethylethyl)-4-methylphenol (Sigma-Aldrich, Warsaw, Poland) and 1 mL of 5% trichloroacetic acid (Sigma-Aldrich, Warsaw, Poland). The mixture was centrifuged at 14,000× *g* for 10 min, after which 750 μL of the supernatant was transferred to a glass tube and combined with 500 μL of 0.6% thiobarbituric acid (Sigma-Aldrich, Warsaw, Poland). The samples were incubated in a water bath at 90 °C for 45 min and immediately cooled on ice. Following a second centrifugation at 4000× *g* for 5 min, 200 μL of the supernatant was transferred to a microplate. Absorbance was recorded at 532 nm using an Infinite M200 Pro microplate reader equipped with the NanoQuant system (Tecan Austria GmbH, Grödig, Austria).

Total antioxidant status was measured using the Total Antioxidant Status assay (Cat. No. NX2331; Randox Laboratories Ltd., Crumlin, UK). The assay is based on the formation of the ABTS^®^ radical cation following incubation of ABTS^®^ with metmyoglobin and hydrogen peroxide. Antioxidants present in the serum suppress radical cation formation, and the resulting decrease in color intensity is proportional to the antioxidant capacity of the sample. Absorbance was measured at 600 nm using an Infinite M200 Pro microplate reader equipped with the NanoQuant system (Tecan Austria GmbH, Grödig, Austria).

Glutathione reductase activity was determined using the Glutathione Reductase assay (Cat. No. GR2608; Randox Laboratories Ltd., Crumlin, UK). The assay is based on the NADPH-dependent reduction in oxidized glutathione (GSSG) to reduced glutathione (GSH). Measurements were performed using an Infinite M200 Pro microplate reader equipped with the NanoQuant system (Tecan Austria GmbH, Grödig, Austria), in accordance with the assay manufacturer’s instructions.

Glutathione peroxidase activity was measured using the Ransel Glutathione Peroxidase assay (Cat. No. RS505; Randox Laboratories Ltd., Crumlin, UK). Enzyme activity was determined on the basis of glutathione oxidation in the presence of cumene hydroperoxide according to the procedure specified by the manufacturer (Randox Laboratories Ltd., Crumlin, UK). Absorbance measurements were performed using an Infinite M200 Pro microplate reader equipped with the NanoQuant system (Tecan Austria GmbH, Grödig, Austria).

Superoxide dismutase activity was determined using the Ransod Superoxide Dismutase assay (Cat. No. SD126; Randox Laboratories Ltd., Crumlin, UK). The method is based on the generation of superoxide radicals by the xanthine–xanthine oxidase system and determination of the inhibitory effect of SOD on the reaction. Measurements were carried out using an Infinite M200 Pro microplate reader equipped with the NanoQuant system (Tecan Austria GmbH, Grödig, Austria), following the manufacturer’s assay procedure.

All serum samples were analyzed in duplicate.

### 4.4. Determination of Milk Physicochemical Composition

The physicochemical composition of individual milk samples was determined at the laboratory of the Warsaw University of Life Sciences (Warsaw, Poland). Milk fat, total protein, lactose, total solids, solids-not-fat, and urea concentrations were measured by Fourier-transform infrared (FTIR) spectrometry using a MilkoScan FT-120 analyser (FOSS Electric A/S, Hillerød, Denmark). Milk pH was determined as part of the physicochemical assessment. Casein content was derived from the total protein content according to the analytical procedure used by the laboratory. Somatic cell count (SCC) was determined by flow cytometry using a Somacount 150 analyser (Bentley Instruments Inc., Chaska, MN, USA). SCC results were expressed as ×10^3^ cells/mL and were subsequently used to classify cows into four experimental categories: <100, 100–199, 200–399, and ≥400 × 10^3^ cells/mL.

### 4.5. Determination of Bioactive Proteins

Milk concentrations of lactoferrin (LF), β-lactoglobulin (β-LG), lysozyme (LZ), and lactoperoxidase (LP) were determined by reversed-phase high-performance liquid chromatography (RP-HPLC). Prior to chromatographic analysis, milk samples were centrifuged at 5000× *g* for 15 min at 4 °C, and the separated fat layer was removed. Casein was subsequently precipitated from the skimmed milk by the addition of 10% acetic acid. Following precipitation, the samples were centrifuged at 14,000× *g* for 15 min, and the resulting supernatant containing the whey protein fraction was collected and passed through a 0.22-μm membrane filter. Chromatographic separation was performed using an Agilent 1100 Series HPLC system (Agilent Technologies, Waldbronn, Germany) equipped with a Jupiter C18 analytical column with a 300 Å pore size (Phenomenex, Torrance, CA, USA). Lactoferrin, α-LA, β-LG, LZ, IgG, IgM, and IgA were identified by comparison with the corresponding certified analytical standards obtained from Sigma-Aldrich (St. Louis, MO, USA). Protein concentrations were calculated by external calibration using calibration curves generated from the respective standards. Protein concentrations were calculated by external calibration using calibration curves generated from the respective standards. Each milk sample was analysed in duplicate.

### 4.6. Determination of Inflammatory Cytokines

The inflammatory profile of milk was characterized by quantitative determination of seven cytokines: interleukin-1β (IL-1β), interleukin-6 (IL-6), interleukin-8 (IL-8), interleukin-10 (IL-10), tumor necrosis factor-α (TNF-α), interleukin-12 (IL-12), and interleukin-17A (IL-17A). Cytokine concentrations were measured using bovine-specific quantitative sandwich enzyme-linked immunosorbent assays (ELISA). Milk intended for cytokine analysis was maintained at 4 °C during transport and processed within 2 h of collection. Samples were initially centrifuged at 5000× *g* for 15 min at 4 °C to separate the lipid fraction. After removal of the upper fat layer, the skimmed milk was carefully recovered without disturbing the sediment and subjected to a second centrifugation at 14,000× *g* for 10 min at 4 °C. This step was used to obtain a clarified milk fraction free of residual particulate material and cellular debris. The resulting supernatant was aliquoted and stored at −80 °C until cytokine determination. Aliquots were thawed once, immediately before analysis, and repeated freeze–thaw cycles were avoided. IL-1β, IL-6, IL-8, IL-10, and TNF-α were quantified using bovine-specific ELISA supplied by MyBioSource Inc. (San Diego, CA, USA): Bovine IL-1β ELISA Kit (Cat. No. MBS703996), Bovine IL-6 ELISA Kit (Cat. No. MBS2020104), Bovine IL-8 ELISA Kit (Cat. No. MBS8105), Bovine IL-10 ELISA Kit (Cat. No. MBS703712), and Bovine TNF-α ELISA Kit (Cat. No. MBS702888). IL-12 and IL-17A were determined using the ELISA Kit for Interleukin 12 (IL-12; Cat. No. SEA059Bo) and ELISA Kit for Interleukin 17A (IL-17A; Cat. No. SEA063Bo), respectively (Cloud-Clone Corp., Wuhan, China). Individual assays were performed in accordance with the analytical procedures specified by the respective manufacturers. Before analysis of the study samples, the suitability of each immunoassay for use with the bovine milk matrix was evaluated. Assay performance was examined using pooled bovine milk by serial dilution and spike-and-recovery testing. Serial dilutions were used to assess dilutional linearity, whereas recovery experiments were performed to evaluate the effect of the milk matrix on analyte quantification. Only assays showing acceptable recovery and linearity within the analytical range specified for the respective assay were used for subsequent determinations. A separate calibration curve was generated on each analytical plate using the recombinant bovine standards supplied with the corresponding ELISA kit. Standards were prepared and analysed according to the manufacturer’s protocol. Cytokine concentrations in the milk samples were calculated from the resulting standard curves using a four-parameter logistic (4-PL) regression model. Absorbance was measured at 450 nm with reference wavelength correction at 620 nm using an Infinite M200 Pro microplate reader equipped with the NanoQuant system (Tecan Austria GmbH, Grödig, Austria). Standards, blanks, internal quality-control samples, and experimental milk samples were analysed in duplicate. The agreement between duplicate measurements was evaluated within each analytical run, and samples for which duplicate determinations differed by more than 10% were reanalysed. Assay precision was monitored throughout the analytical period using internal quality-control samples included on each plate. The intra-assay coefficient of variation (CV) remained below 10%, whereas the inter-assay CV did not exceed 15% for any of the cytokines included in the analysis. IL-8 concentrations were expressed as ng/mL, whereas the remaining cytokines were expressed as pg/mL.

### 4.7. Determination of Cis-9,Trans-11 Conjugated Linoleic Acid

The cis-9,trans-11 isomer of conjugated linoleic acid (CLA c9,t11) was determined in milk fat by gas chromatography following conversion of fatty acids to their corresponding fatty acid methyl esters (FAME). Fatty acid methyl esters were prepared by transesterification according to PN-EN ISO 5509:2000 [42]. Chromatographic separation was carried out using an Agilent 7890 gas chromatograph (Agilent Technologies, Waldbronn, Germany) equipped with a Varian Select FAME capillary column (Varian Inc., Palo Alto, CA, USA). The oven temperature was initially maintained at 130 °C for 1 min and subsequently increased to 170 °C at a rate of 6.5 °C/min. The temperature was then raised from 170 to 215 °C at 2.75 °C/min and maintained at 215 °C for 12 min. In the final stage of the temperature programme, the oven temperature was increased from 215 to 230 °C at 20 °C/min and held at 230 °C for 3 min. Helium was used as the carrier gas at a linear velocity of 25 cm/s. The injector and detector temperatures were maintained at 240 and 300 °C, respectively. Identification of CLA c9,t11 was based on comparison of the retention time of the chromatographic peak obtained from milk FAME with that of the corresponding methyl ester present in a conjugated linoleic acid methyl ester isomer reference mixture (Linoleic acid, conjugated methyl ester isomer mix, Product No. 05632; Supelco/Sigma-Aldrich, Bellefonte, PA, USA). The reference mixture was analysed under the same chromatographic conditions as the milk FAME samples. The cis-9,trans-11 CLA isomer was identified as methyl 9-cis,11-trans-octadecadienoate (C18:2 c9,t11). The relative content of CLA c9,t11 was calculated from the area of the corresponding chromatographic peak and expressed as a percentage of total identified fatty acids. Each milk sample was analysed in duplicate.

### 4.8. Calculation of the Pro-Inflammatory Cytokine Index and Definition of the Inflammatory Phenotype

A Pro-inflammatory Cytokine Index (PCI) was calculated to provide an integrated measure of the milk pro-inflammatory cytokine response. The index comprised IL-1β, IL-6, IL-8, TNF-α, IL-12, and IL-17A. Because the individual cytokines differed in their absolute concentrations and analytical scales, concentrations were standardized across the study population before calculation of the index. For each cytokine, a standardized value (*z*-score) was calculated as: z = (x − μ)/σ; where *x* denotes the individual cytokine concentration, μ the population mean, and σ the corresponding population standard deviation. The PCI for each cow was subsequently calculated as the arithmetic mean of the standardized values of the six pro-inflammatory cytokines: PCI = [z(IL-1β) + z(IL-6) + z(IL-8) + z(TNF-α) + z(IL-12) + z(IL-17A)]/6. This calculation gave equal weight to each cytokine irrespective of its original concentration range. Increasing PCI values reflected a progressively stronger overall pro-inflammatory cytokine response. IL-10 was not incorporated into the PCI because of its predominantly anti-inflammatory activity and was evaluated separately. For classification analyses, an inflammatory phenotype was defined independently of SCC and PCI. Classification was based on the concordance of the cytokine response rather than on the concentration of a single mediator. The six pro-inflammatory cytokines were standardized relative to their distributions in the study population, and IL-10 was standardized separately in the same manner. Cows were classified as inflammatory-phenotype positive when at least four of the six pro-inflammatory cytokines (IL-1β, IL-6, IL-8, TNF-α, IL-12, and IL-17A) had standardized values above the population mean (*z* > 0) and IL-10 showed a standardized value below the population mean (*z* < 0). Animals not meeting both criteria were classified as inflammatory-phenotype negative. Requiring concordant changes in multiple pro-inflammatory mediators together with a reduction in the anti-inflammatory component was intended to distinguish a coordinated inflammatory response from an isolated increase in a single cytokine. Neither SCC nor PCI contributed to assignment of inflammatory phenotype status. The resulting binary classification was used as the reference outcome for evaluation of alternative SCC thresholds and for ROC analyses of individual cytokines, PCI, and integrated biomarker models. Because the inflammatory phenotype was derived from the same cytokine panel used to calculate PCI and to evaluate individual cytokines, ROC analyses involving these inflammatory variables were interpreted as measures of internal correspondence with the study-derived phenotype rather than as independent diagnostic validation. The phenotype was therefore used as an analytical reference for characterizing coordinated inflammatory activity within the study population, and not as an externally validated diagnostic endpoint.

### 4.9. Statistical Analysis

Statistical analyses were performed using IBM SPSS Statistics, version 29.0 (IBM Corp., Armonk, NY, USA) and MetaboAnalyst 6.0 (McGill University, Montreal, QC, Canada). The individual cow was considered the experimental unit. Data were checked for completeness and extreme observations before analysis. Distributional assumptions for continuous variables were evaluated using the Shapiro–Wilk test, supplemented by inspection of the corresponding distributions. Homogeneity of variances was assessed using Levene’s test. Variables showing marked departures from normality were transformed where necessary. Somatic cell count was log10-transformed when included as a continuous predictor in parametric regression analyses because of its right-skewed distribution. For rank-based Spearman correlation analysis, SCC was analysed on its original scale. Continuous variables are reported as mean ± standard deviation (SD), unless otherwise specified.

Cows were classified into four SCC categories: <100, 100–199, 200–399, and ≥400 × 10^3^ cells/mL. Differences among these categories were examined by one-way analysis of variance (ANOVA). When the overall effect of SCC category was significant, Tukey’s honestly significant difference (HSD) test was used for post-hoc comparisons. Means not sharing a common superscript letter were considered significantly different at *p* < 0.05. In addition to the overall comparison, three a priori contrasts were specified between consecutive SCC categories (<100 vs. 100–199, 100–199 vs. 200–399, and 200–399 vs. ≥400 × 10^3^ cells/mL). These prespecified contrasts were analysed separately to characterize between-group changes across consecutive SCC categories and were considered complementary to, rather than determinative of, the continuous breakpoint analysis described below.

Associations between SCC and biological variables were examined using Spearman’s rank correlation coefficients (ρ). Spearman’s correlation was selected because several biomarker relationships with SCC were monotonic but not necessarily linear across the full SCC range. Correlations were calculated using the original SCC values, as rank-based analysis does not require transformation of the predictor. SCC was correlated with milk production and composition traits, inflammatory cytokines, the Pro-inflammatory Cytokine Index (PCI), mammary innate-defense proteins, metabolic and hepatic biomarkers, oxidative stress indices, BCS, and milk yield. Correlation coefficients (ρ) are reported together with the corresponding two-sided *p*-values.

Multivariable analyses incorporated factors that could contribute to variation independently of mammary inflammatory status. Year of sampling was included to account for temporal variation associated with the two-year study period. Days in milk (DIM), lactation number, BCS, and body weight were included as cow-level covariates. Their inclusion was intended to account for variation related to stage of lactation, parity, body reserves, and animal size.

Binary logistic regression was used to identify variables independently associated with SCC ≥ 200 × 10^3^ cells/mL. SCC was entered as a binary outcome (<200 vs. ≥200 × 10^3^ cells/mL), with SCC ≥ 200 × 10^3^ cells/mL coded as the event. Candidate predictors represented inflammatory, metabolic, oxidative, production-related, and animal-level domains. Days in milk (DIM), lactation number, BCS, year of sampling, and body weight were evaluated as potential cow-level covariates to account for variation related to stage of lactation, parity, body reserves, temporal variation, and animal size. Year of sampling and body weight were not retained in the final model because their inclusion did not materially alter the estimated associations. Multicollinearity among predictors was assessed using tolerance statistics and variance inflation factors (VIFs), with VIF > 5 or tolerance <0.20 considered indicative of potentially problematic collinearity. The assumption of linearity in the logit was evaluated for continuous predictors using the Box–Tidwell procedure. Model coefficients were exponentiated and are reported as adjusted odds ratios (OR) with 95% confidence intervals (CI). For continuous predictors, ORs were expressed for predefined increments selected to provide biologically interpretable effect estimates. Overall model calibration was assessed using the Hosmer–Lemeshow goodness-of-fit test, whereas model explanatory capacity was summarized using Nagelkerke R^2^. Discrimination was evaluated by the area under the receiver operating characteristic curve (AUC) with 95% CI.

Breakpoint analysis was performed using segmented linear regression to determine whether the relationship between SCC and individual biological responses changed along the continuous SCC distribution. Because SCC was right-skewed, log10-transformed SCC was used as the predictor. Separate continuous two-segment regression models with a single unknown change point were fitted for IL-8, IL-1β, IL-6, TNF-α, IL-12, IL-17A, IL-10, PCI, lysozyme, lactoperoxidase, MDA, and TAS. Continuity of the regression function was imposed at the estimated breakpoint. The change point was estimated directly from the data and was not constrained to coincide with any predefined SCC category or with the conventional threshold of 200 × 10^3^ cells/mL. For each biomarker, the segmented model was compared with the corresponding single-slope linear model to evaluate whether inclusion of a change point improved model fit. Regression slopes were estimated separately below and above the breakpoint. The stability and uncertainty of breakpoint estimates were assessed by non-parametric bootstrap resampling with 2000 iterations. For each bootstrap sample, the segmented model was refitted and the change point was re-estimated without constraining its location. The empirical bootstrap distributions were used to derive 95% confidence intervals for the estimated breakpoints. Breakpoint estimates obtained on the log10 scale were subsequently back-transformed and are reported as SCC × 10^3^ cells/mL. Breakpoint estimation was conducted independently of all subsequent SCC threshold-based analyses; therefore, the location of the estimated change points was not determined by the predefined 200 × 10^3^ cells/mL cut-off.

Receiver operating characteristic analysis was used to quantify the ability of individual inflammatory variables to discriminate between cows with and without the inflammatory phenotype. Separate ROC curves were constructed for IL-1β, IL-6, IL-8, IL-10, TNF-α, IL-12, IL-17A, and PCI. Discriminatory performance was expressed as the area under the ROC curve (AUC) with 95% CI. For each marker, the optimal cut-off was selected by maximizing the Youden index, and sensitivity and specificity were calculated at that cut-off. The performance of combinations of biomarkers was subsequently examined using multivariable logistic models from which individual predicted probabilities were obtained for ROC analysis. Five models were evaluated. Model 1 contained IL-8 alone. Model 2 included IL-8, IL-6, and TNF-α. Model 3 comprised IL-8, IL-6, TNF-α, IL-17A, and IL-10. Model 4 combined PCI with MDA and TAS, whereas Model 5 included PCI, MDA, TAS, NEFA, and BCS. ROC curves were constructed from the predicted probabilities generated by each model. Model discrimination was characterized by AUC with 95% CI, sensitivity, and specificity, with the operating point determined using the Youden criterion.

Internal validation of model discrimination was performed using non-parametric bootstrap resampling with 2000 iterations. In each bootstrap iteration, the complete model was refitted and its discriminatory performance was evaluated both in the bootstrap sample and in the original dataset. Optimism was estimated as the mean difference between the AUC obtained in the bootstrap sample and the AUC obtained when the corresponding bootstrap-fitted model was evaluated in the original dataset. The optimism-corrected AUC was calculated by subtracting the mean bootstrap optimism from the apparent AUC estimated in the original study population. Bootstrap resampling was performed independently for PCI and for the sequential inflammatory–oxidative and inflammatory–oxidative–metabolic models. To determine whether the addition of oxidative and metabolic variables provided incremental discriminatory information beyond the inflammatory component, correlated ROC curves were compared using DeLong’s method. Sequential comparisons were performed between PCI alone and the inflammatory–oxidative model comprising PCI, MDA, and TAS, and between the inflammatory–oxidative model and the fully integrated inflammatory–oxidative–metabolic model comprising PCI, MDA, TAS, NEFA, and BCS. Differences in AUC (ΔAUC) were calculated for each comparison and are presented with 95% confidence intervals and two-sided *p*-values. Internal validation and pairwise comparison of the sequential biomarker models are summarized in Table 17.

A complementary analysis was performed after dichotomizing the population at an SCC of 200 × 10^3^ cells/mL. Cows with SCC < 200 × 10^3^ cells/mL were compared with cows with SCC ≥ 200 × 10^3^ cells/mL for selected inflammatory, oxidative, metabolic, animal-level, and production-related variables. Relative change was calculated with the SCC < 200 × 10^3^ cells/mL group as the reference. The magnitude of each between-group difference was additionally quantified using Cohen’s *d*, calculated as the difference between group means divided by the pooled SD. The algebraic sign of Cohen’s *d* was retained to indicate the direction of the difference.

Because multiple biomarkers were evaluated within biologically related domains, the potential inflation of type I error arising from multiple comparisons was addressed using the Benjamini–Hochberg false discovery rate (FDR) procedure. FDR adjustment was applied separately within predefined families of inflammatory, mammary innate-defense, oxidative/antioxidant, metabolic/hepatic, and milk production/composition variables. This domain-specific approach was used to preserve the biological structure of the hypotheses while controlling the expected proportion of false discoveries within each family of related comparisons. Both nominal *p*-values and FDR-adjusted *p*-values (*q*-values) were retained, and *q* < 0.05 was considered statistically significant after correction for multiple testing. Nominal *p*-values from the Spearman correlation analyses were likewise adjusted using the Benjamini–Hochberg procedure across the set of correlations evaluated. FDR adjustment was not applied to the prespecified segmented breakpoint models, coefficients of the multivariable logistic regression model, bootstrap internal validation, or the two prespecified sequential DeLong comparisons, as these were treated as distinct model-based analyses rather than families of parallel univariate biomarker tests. Effect estimates and 95% confidence intervals were interpreted together with the corresponding probability values. All statistical tests were two-sided; *p* < 0.05 was considered statistically significant for analyses not subject to FDR adjustment, whereas *q* < 0.05 was used for FDR-adjusted analyses.

## 5. Conclusions

The present study indicates that, in clinically healthy cows without evidence of intramammary infection, increasing SCC was accompanied by a coordinated biological response that became particularly evident in the region around 200 × 10^3^ cells/mL. The principal evidence for this transition was the close convergence of independently estimated breakpoints for 12 inflammatory, innate-defense, and oxidative biomarkers, all of which fell between 198 and 228 × 10^3^ cells/mL. IL-8 was the marker most closely associated with this transition and an estimated breakpoint of 198 × 10^3^ cells/mL. The transition was characterized by a shift toward pro-inflammatory cytokine activity, changes in selected mammary innate-defense components, deterioration of oxidative balance, and concomitant metabolic and production-related alterations. Categorical comparisons and threshold analyses were consistent with this continuously derived pattern; 200 × 10^3^ cells/mL provided the most favorable balance between sensitivity and specificity for identifying the study-defined inflammatory phenotype. These findings do not establish 200 × 10^3^ cells/mL as a universal diagnostic threshold for mastitis or intramammary infection but rather identify this value as a biologically informative transition region in mammary inflammatory status, accompanied by systemic oxidative and metabolic changes. Independent prospective validation across herds, lactation stages, parities, and production systems is required to determine the generalizability of this transition.

## Figures and Tables

**Table 1:** Milk production and composition according to SCC category.

Parameter	SCC < 100 (*n* = 285)	SCC 100–199 (*n* = 135)	SCC 200–399 (*n* = 90)	SCC ≥ 400 (*n* = 70)	*p*-Value	FDR-Adjusted *q*-Value
Milk yield, kg/day	34.1 ± 7.7 ^a^	33.6 ± 7.8 ^a^	31.7 ± 8.0 ^b^	28.9 ± 8.4 ^c^	<0.001	<0.002
Fat, %	3.86 ± 0.57 ^a^	3.79 ± 0.60 ^a^	3.62 ± 0.68 ^b^	3.40 ± 0.77 ^c^	<0.001	<0.002
Protein, %	3.39 ± 0.36	3.40 ± 0.37	3.44 ± 0.39	3.48 ± 0.42	0.067	0.067
CLA c9,t11, %	0.66 ± 0.27 ^a^	0.65 ± 0.27 ^a^	0.61 ± 0.25 ^ab^	0.56 ± 0.23 ^b^	0.006	0.008

Values are presented as mean ± SD. SCC, somatic cell count (×10^3^ cells/mL); CLA c9,t11, cis-9, trans-11 conjugated linoleic acid. Different superscript letters within a row indicate significant differences (*p* < 0.05). FDR-adjusted *q*-values are reported for multiple comparisons.

**Table 2:** Inflammatory cytokine concentrations according to SCC category.

Cytokine	SCC < 100 (*n* = 285)	SCC 100–199 (*n* = 135)	SCC 200–399 (*n* = 90)	SCC ≥ 400 (*n* = 70)	*p*-Value	FDR-Adjusted *q*-Value
IL-1β, pg/mL	18.2 ± 8.1 ^a^	19.5 ± 8.9 ^a^	31.8 ± 14.2 ^b^	48.6 ± 21.3 ^c^	<0.001	<0.001
IL-6, pg/mL	24.5 ± 11.4 ^a^	25.8 ± 12.0 ^a^	41.6 ± 19.1 ^b^	65.3 ± 29.8 ^c^	<0.001	<0.001
IL-8, ng/mL	0.69 ± 0.31 ^a^	0.78 ± 0.39 ^a^	2.42 ± 1.51 ^b^	7.68 ± 5.20 ^c^	<0.001	<0.001
IL-10, pg/mL	29.8 ± 12.4 ^a^	30.4 ± 13.0 ^a^	26.1 ± 11.5 ^b^	21.9 ± 10.2 ^c^	<0.001	<0.001
TNF-α, pg/mL	20.1 ± 9.3 ^a^	21.4 ± 9.7 ^a^	34.9 ± 15.8 ^b^	52.7 ± 24.6 ^c^	<0.001	<0.001
IL-12, pg/mL	15.8 ± 7.0 ^a^	16.6 ± 7.5 ^a^	23.9 ± 10.8 ^b^	31.7 ± 14.2 ^c^	<0.001	<0.001
IL-17A, pg/mL	11.4 ± 5.2 ^a^	12.1 ± 5.7 ^a^	20.8 ± 9.7 ^b^	33.9 ± 15.8 ^c^	<0.001	<0.001

Values are presented as mean ± SD. SCC, somatic cell count (×10^3^ cells/mL); IL, interleukin; TNF-α, tumor necrosis factor alpha. Different superscript letters within a row indicate significant differences (*p* < 0.05). FDR-adjusted *q*-values are reported for multiple comparisons.

**Table 3:** Pro- and anti-inflammatory balance.

Index	SCC < 100 (*n* = 285)	SCC 100–199 (*n* = 135)	SCC 200–399 (*n* = 90)	SCC ≥ 400 (*n* = 70)	*p*-Value	FDR-Adjusted *q*-Value
IL-6/IL-10	0.82 ^a^	0.85 ^a^	1.59 ^b^	2.98 ^c^	<0.001	<0.001
TNF-α/IL-10	0.67 ^a^	0.70 ^a^	1.34 ^b^	2.41 ^c^	<0.001	<0.001
IL-1β/IL-10	0.61 ^a^	0.64 ^a^	1.22 ^b^	2.22 ^c^	<0.001	<0.001
IL-17A/IL-10	0.38 ^a^	0.40 ^a^	0.80 ^b^	1.55 ^c^	<0.001	<0.001
PCI	−0.46 ^a^	−0.39 ^a^	0.54 ^b^	1.47 ^c^	<0.001	<0.001

SCC, somatic cell count (×10^3^ cells/mL); IL, interleukin; TNF-α, tumor necrosis factor alpha; PCI, Pro-inflammatory Cytokine Index. Different superscript letters within a row indicate significant differences (*p* < 0.05). FDR-adjusted *q*-values are reported for multiple comparisons.

**Table 4:** Mammary gland bioactive proteins according to SCC category.

Parameter	SCC < 100 (*n* = 285)	SCC 100–199 (*n* = 135)	SCC 200–399 (*n* = 90)	SCC ≥ 400 (*n* = 70)	*p*-Value	FDR-Adjusted *q*-Value
Lactoferrin, mg/L	168.4 ± 73.2 ^a^	176.9 ± 78.6 ^a^	249.7 ± 105.3 ^b^	346.8 ± 142.1 ^c^	<0.001	<0.001
Lysozyme, µg/L	5.9 ± 2.5 ^a^	6.2 ± 2.7 ^a^	9.1 ± 3.8 ^b^	13.4 ± 5.6 ^c^	<0.001	<0.001
Lactoperoxidase, mg/L	1.21 ± 0.46 ^a^	1.25 ± 0.49 ^a^	1.69 ± 0.63 ^b^	2.18 ± 0.82 ^c^	<0.001	<0.001
β-lactoglobulin, g/L	2.84 ± 1.16 ^a^	2.96 ± 1.21 ^a^	4.12 ± 1.64 ^b^	5.78 ± 2.27 ^c^	<0.001	<0.001

Values are presented as mean ± SD. SCC, somatic cell count (×10^3^ cells/mL). Different superscript letters within a row indicate significant differences (*p* < 0.05). FDR-adjusted *q*-values are reported for multiple comparisons.

**Table 5:** Metabolic and hepatic profile according to SCC category.

Parameter	SCC < 100 (*n* = 285)	SCC 100–199 (*n* = 135)	SCC 200–399 (*n* = 90)	SCC ≥ 400 (*n* = 70)	*p*-Value	FDR-Adjusted *q*-Value
NEFA, mmol/L	0.34 ± 0.15 ^a^	0.36 ± 0.16 ^a^	0.45 ± 0.20 ^b^	0.55 ± 0.24 ^c^	<0.001	<0.001
BHBA, mmol/L	0.71 ± 0.29 ^a^	0.74 ± 0.31 ^a^	0.86 ± 0.37 ^b^	0.98 ± 0.43 ^c^	<0.001	<0.001
Glucose, mmol/L	3.46 ± 0.47 ^a^	3.43 ± 0.48 ^a^	3.32 ± 0.51 ^b^	3.18 ± 0.55 ^c^	<0.001	<0.001
AST, U/L	76.8 ± 17.9 ^a^	78.1 ± 18.4 ^a^	83.9 ± 20.7 ^b^	90.6 ± 23.8 ^c^	<0.001	<0.001
GGT, U/L	23.7 ± 6.9 ^a^	24.2 ± 7.1 ^a^	26.8 ± 7.9 ^b^	29.5 ± 8.8 ^c^	<0.001	<0.001

Values are presented as mean ± SD. SCC, somatic cell count (×10^3^ cells/mL); NEFA, non-esterified fatty acids; BHBA, β-hydroxybutyrate; AST, aspartate aminotransferase; GGT, γ-glutamyl transferase. Different superscript letters within a row indicate significant differences (*p* < 0.05). FDR-adjusted *q*-values are reported for multiple comparisons.

**Table 6:** Oxidative status according to SCC category.

Parameter	SCC < 100 (*n* = 285)	SCC 100–199 (*n* = 135)	SCC 200–399 (*n* = 90)	SCC ≥ 400 (*n* = 70)	*p*-Value	FDR-Adjusted *q*-Value
MDA, µmol/L	28.1 ± 7.0 ^a^	28.9 ± 7.6 ^a^	33.7 ± 9.4 ^b^	38.5 ± 11.7 ^c^	<0.001	0.0005
TAS, mmol/L	1.86 ± 1.18 ^a^	1.79 ± 1.16 ^a^	1.43 ± 1.10 ^b^	1.11 ± 0.95 ^c^	<0.001	0.0005
SOD, U/L	237 ± 69 ^a^	241 ± 72 ^a^	258 ± 86 ^b^	276 ± 104 ^b^	0.002	0.0033
GPx, U/L	476 ± 196	480 ± 202	471 ± 205	465 ± 211	0.713	0.713
GluRed, U/L	81 ± 35 ^a^	83 ± 36 ^a^	89 ± 39 ^ab^	96 ± 43 ^b^	0.018	0.0225

Values are presented as mean ± SD. SCC, somatic cell count (×10^3^ cells/mL); MDA, malondialdehyde; TAS, total antioxidant status; SOD, superoxide dismutase; GPx, glutathione peroxidase; GluRed, glutathione reductase. Different superscript letters within a row indicate significant differences (*p* < 0.05). FDR-adjusted *q*-values are reported for multiple comparisons.

**Table 7:** Planned contrasts around the 200 × 10^3^ cells/mL threshold.

Variable	<100 vs. 100–199 *p*	FDR *q*	100–199 vs. 200–399 *p*	FDR *q*	200–399 vs. ≥400 *p*	FDR *q*
IL-1β	0.318	0.539	<0.001	<0.001	<0.001	<0.001
IL-6	0.291	0.539	<0.001	<0.001	<0.001	<0.001
IL-8	0.214	0.539	<0.001	<0.001	<0.001	<0.001
IL-10	0.684	0.684	0.002	0.002	0.004	0.004
TNF-α	0.276	0.539	<0.001	<0.001	<0.001	<0.001
IL-12	0.412	0.549	<0.001	<0.001	0.002	0.002
IL-17A	0.337	0.539	<0.001	<0.001	<0.001	<0.001
PCI	0.527	0.602	<0.001	<0.001	<0.001	<0.001
Lysozyme	0.463	0.572	<0.001	<0.001	<0.001	<0.001
Lactoperoxidase	0.572	0.572	<0.001	<0.001	<0.001	<0.001
MDA	0.496	0.496	<0.001	<0.001	0.003	0.006
TAS	0.381	0.496	<0.001	<0.001	0.006	0.006
NEFA	0.438	0.438	0.003	0.003	0.008	0.008
BCS	0.603	0.603	0.011	0.011	0.019	0.019

SCC, somatic cell count; IL, interleukin; TNF-α, tumor necrosis factor alpha; PCI, Pro-inflammatory Cytokine Index; MDA, malondialdehyde; TAS, total antioxidant status; NEFA, non-esterified fatty acids; BCS, body condition score. Values represent nominal *p*-values and corresponding FDR-adjusted *q*-values for prespecified contrasts between adjacent SCC categories.

**Table 8:** Spearman rank correlations between SCC and biological markers.

Marker	Spearman’s ρ	*p*-Value	FDR-Adjusted *q*-Value
IL-8	0.77	<0.001	<0.001
PCI	0.74	<0.001	<0.001
IL-6	0.65	<0.001	<0.001
TNF-α	0.63	<0.001	<0.001
IL-1β	0.62	<0.001	<0.001
IL-17A	0.60	<0.001	<0.001
Lysozyme	0.59	<0.001	<0.001
Lactoperoxidase	0.61	<0.001	<0.001
IL-12	0.49	<0.001	<0.001
MDA	0.51	<0.001	<0.001
NEFA	0.47	<0.001	<0.001
TAS	−0.44	<0.001	<0.001
AST	0.42	<0.001	<0.001
BCS	−0.41	<0.001	<0.001
BHBA	0.39	<0.001	<0.001
GGT	0.38	<0.001	<0.001
IL-10	−0.34	<0.001	<0.001
Milk yield	−0.52	<0.001	<0.001

SCC, somatic cell count; ρ, Spearman’s rank correlation coefficient; IL, interleukin; TNF-α, tumor necrosis factor alpha; PCI, Pro-inflammatory Cytokine Index; NEFA, non-esterified fatty acids; BHBA, β-hydroxybutyrate; AST, aspartate aminotransferase; GGT, gamma-glutamyl transferase; MDA, malondialdehyde; TAS, total antioxidant status; BCS, body condition score. Nominal *p*-values were adjusted for multiple testing using the Benjamini–Hochberg false discovery rate (FDR) procedure across the 18 correlations included in the analysis. A *q*-value < 0.05 was considered statistically significant after correction for multiple comparisons.

**Table 9:** Multivariable logistic regression model for SCC ≥ 200 × 10^3^ cells/mL.

Predictor	Adjusted OR	95% CI	*p*-Value
IL-8, per 1 ng/mL	1.84	1.60–2.13	<0.001
IL-6, per 10 pg/mL	1.31	1.16–1.49	<0.001
IL-17A, per 10 pg/mL	1.28	1.09–1.51	0.003
IL-10, per 10 pg/mL	0.81	0.69–0.95	0.010
NEFA, per 0.5 mmol/L	1.42	1.11–1.82	0.006
MDA, per 10 µmol/L	1.29	1.07–1.56	0.008
BCS, per 1 point	0.71	0.53–0.95	0.021
DIM, per day	1.01	0.98–1.04	0.477
Lactation number	1.09	0.75–1.59	0.648
Milk yield, per kg/d	0.98	0.96–1.01	0.146

Results are presented as adjusted odds ratios (OR) with 95% confidence intervals (CI) from the final multivariable binary logistic regression model for SCC ≥ 200 × 10^3^ cells/mL. Continuous predictors were scaled as indicated in the table. OR >1 indicates higher adjusted odds, whereas OR <1 indicates lower adjusted odds of SCC ≥ 200 × 10^3^ cells/mL.

**Table 10:** Estimated SCC breakpoints for inflammatory, mammary innate-defense, and oxidative stress biomarkers.

Marker	Estimated Breakpoint, ×10^3^ Cells/mL	Bootstrap 95% CI	Slope Below Breakpoint	Slope Above Breakpoint	*p* for Change in Slope
IL-8, ng/mL	198	174–226	+0.05	+1.31	<0.001
IL-1β, pg/mL	216	184–253	+0.72	+7.84	<0.001
IL-6, pg/mL	207	179–241	+0.81	+10.26	<0.001
TNF-α, pg/mL	221	188–260	+0.68	+8.91	<0.001
IL-12, pg/mL	228	191–271	+0.41	+4.52	0.002
IL-17A, pg/mL	204	176–238	+0.39	+5.67	<0.001
IL-10, pg/mL	219	184–260	+0.18	−3.21	0.003
PCI	205	181–233	+0.04	+0.46	<0.001
Lysozyme, µg/L	218	184–258	+0.31	+5.18	<0.001
Lactoperoxidase, mg/L	211	181–249	+0.006	+0.086	<0.001
MDA, µmol/L	226	190–269	+0.29	+3.04	<0.001
TAS, mmol/L	214	180–254	−0.02	−0.23	0.002

Breakpoints were estimated using continuous segmented linear regression with log10-transformed SCC as the predictor. Estimates were obtained independently of predefined SCC categories. Breakpoint uncertainty was assessed by non-parametric bootstrap resampling. Slopes represent the estimated change in each biomarker per 0.1-unit increase in log10(SCC) below and above the estimated breakpoint. The *p*-value refers to the change in slope between the two segments. SCC, somatic cell count; IL, interleukin; TNF-α, tumor necrosis factor alpha; PCI, Pro-inflammatory Cytokine Index; MDA, malondialdehyde; TAS, total antioxidant status.

**Table 11:** Classification performance of alternative SCC thresholds for the inflammatory phenotype.

SCC Threshold × 10^3^/mL	Sensitivity	Specificity	PPV	NPV	Youden Index
≥100	94.0%	54.0%	51.5%	94.8%	0.48
≥200	88.0%	82.0%	68.9%	93.4%	0.70
≥300	73.0%	88.0%	73.7%	87.6%	0.61
≥400	58.0%	93.0%	80.4%	81.7%	0.51

SCC, somatic cell count; PPV, positive predictive value; NPV, negative predictive value. Sensitivity, specificity, PPV, and NPV were calculated for the identification of the inflammatory phenotype. The Youden index was calculated as sensitivity + specificity − 1, with higher values indicating a better balance between sensitivity and specificity.

**Table 12:** ROC performance of inflammatory biomarkers for identifying the inflammatory phenotype.

Biomarker	AUC	95% CI	Sensitivity	Specificity
IL-1β	0.842	0.807–0.875	78.1%	77.4%
IL-6	0.875	0.843–0.904	81.3%	79.6%
IL-8	0.934	0.910–0.954	88.8%	86.5%
IL-10	0.721	0.680–0.760	66.9%	67.5%
TNF-α	0.861	0.827–0.891	79.4%	80.1%
IL-12	0.789	0.751–0.824	72.5%	73.4%
IL-17A	0.873	0.841–0.902	81.9%	79.2%
PCI	0.951	0.930–0.968	90.0%	88.2%

ROC, receiver operating characteristic; AUC, area under the ROC curve; CI, confidence interval; IL, interleukin; TNF-α, tumor necrosis factor alpha; PCI, Pro-inflammatory Cytokine Index. ROC analyses were performed for the identification of the inflammatory phenotype. AUC values are presented with 95% CI. Higher AUC values indicate greater correspondence with the study-derived inflammatory phenotype; these estimates should not be interpreted as independent diagnostic validation.

**Table 13:** Integrated models for inflammatory phenotype.

Model	Variables	Apparent AUC	Bootstrap Optimism	Optimism-Corrected AUC	Bootstrap 95% CI	Sensitivity	Specificity
PCI alone	PCI	0.951	0.006	0.945	0.921–0.963	90.0%	88.2%
Model 1	IL-8	0.934	0.004	0.930	0.904–0.949	88.8%	86.5%
Model 2	IL-8 + IL-6 + TNF-α	0.949	0.006	0.943	0.919–0.960	89.4%	88.0%
Model 3	IL-8 + IL-6 + TNF-α + IL-17A + IL-10	0.959	0.008	0.951	0.929–0.966	91.3%	88.7%
Model 4	PCI + MDA + TAS	0.963	0.009	0.954	0.932–0.970	91.9%	89.2%
Model 5	PCI + MDA + TAS + NEFA + BCS	0.967	0.013	0.954	0.931–0.971	92.5%	89.5%

AUC, area under the receiver operating characteristic curve; PCI, Pro-inflammatory Cytokine Index; IL, interleukin; TNF-α, tumor necrosis factor alpha; MDA, malondialdehyde; TAS, total antioxidant status; NEFA, non-esterified fatty acids; BCS, body condition score. Apparent AUC represents discrimination estimated in the original study population. Internal validation was performed using 2000 non-parametric bootstrap resamples. Bootstrap optimism was calculated as the mean difference between model performance in the bootstrap sample and performance of the same fitted model when evaluated in the original dataset. Optimism-corrected AUC was calculated by subtracting mean bootstrap optimism from the apparent AUC. Bootstrap 95% confidence intervals refer to the internally validated discrimination estimates. Sensitivity and specificity are reported at the operating point selected by the Youden criterion in the original dataset.

**Table 14:** Comparison of biological characteristics below and above the SCC threshold of 200 × 10^3^ cells/mL.

Marker	SCC < 200	SCC ≥ 200	Relative Change	Cohen’s d	*p*-Value
IL-8, ng/mL	0.72 ± 0.34	4.72 ± 4.46	+556.7%	1.70	<0.001
IL-6, pg/mL	24.92 ± 11.60	51.97 ± 26.99	+108.6%	1.57	<0.001
IL-1β, pg/mL	18.62 ± 8.38	39.15 ± 19.48	+110.3%	1.65	<0.001
TNF-α, pg/mL	20.52 ± 9.44	42.69 ± 21.93	+108.1%	1.58	<0.001
IL-17A, pg/mL	11.63 ± 5.37	26.53 ± 14.27	+128.2%	1.70	<0.001
IL-10, pg/mL	29.99 ± 12.58	24.26 ± 11.12	−19.1%	−0.47	<0.001
MDA, µmol/L	28.36 ± 7.20	35.80 ± 10.70	+26.2%	0.90	<0.001
TAS, mmol/L	1.84 ± 1.17	1.29 ± 1.05	−29.8%	−0.48	<0.001
NEFA, mmol/L	0.36 ± 0.28	0.59 ± 0.44	+64.1%	0.70	<0.001
BCS	3.29 ± 0.43	2.98 ± 0.53	−9.5%	−0.68	<0.001
Milk yield, kg/d	33.94 ± 7.73	30.48 ± 8.27	−10.2%	−0.44	<0.001

Values are presented as mean ± SD. SCC, somatic cell count; IL, interleukin; TNF-α, tumor necrosis factor alpha; MDA, malondialdehyde; TAS, total antioxidant status; NEFA, non-esterified fatty acids; BCS, body condition score. Relative change was calculated using the SCC < 200 × 10^3^ cells/mL group as the reference. Cohen’s d represents the standardized effect size, with positive values indicating higher and negative values indicating lower values in cows with SCC ≥ 200 × 10^3^ cells/mL. The *p*-values indicate differences between the SCC < 200 and ≥200 × 10^3^ cells/mL groups.

**Table 15:** Characteristics of cows according to somatic cell count category.

Variable	SCC < 100	SCC 100–199	SCC 200–399	SCC ≥ 400	*p*-Value
*n*	285	135	90	70	—
% of population	49.1	23.3	15.5	12.1	—
BCS	3.30 ± 0.43 ^a^	3.26 ± 0.44 ^a^	3.08 ± 0.49 ^b^	2.84 ± 0.55 ^c^	<0.001
DIM, days	54.7 ± 5.6	54.9 ± 5.5	55.2 ± 5.8	55.5 ± 5.9	0.631
Body weight, kg	639 ± 52 ^a^	634 ± 53 ^a^	625 ± 54 ^ab^	612 ± 55 ^b^	0.008
Milk yield, kg/d	34.1 ± 7.7 ^a^	33.6 ± 7.8 ^a^	31.7 ± 8.0 ^b^	28.9 ± 8.4 ^c^	<0.001

Continuous variables are presented as mean ± SD and categorical variables as percentages. SCC, somatic cell count (×10^3^ cells/mL); BCS, body condition score; DIM, days in milk. Within a row, means with different superscript letters differ significantly (*p* < 0.05). The *p*-values indicate the overall effect of SCC category; “—” indicates that a *p*-value was not applicable or not separately reported.

**Table 16:** Ingredient composition, feeding characteristics, and nutritional and production-related parameters of the total mixed ration (TMR) offered to dairy cows during the study period.

Item	Value	Unit
**Ingredient composition**		
Maize silage	11.05	kg DM/d
Alfalfa silage	3.50	kg DM/d
Ensiled maize grain	2.50	kg DM/d
Soybean meal	2.50	kg DM/d
Rapeseed meal	2.10	kg DM/d
Pasture ground chalk	0.20	kg DM/d
Salt	0.05	kg DM/d
Magnesium oxide	0.06	kg DM/d
**Feeding and nutritional characteristics**		
Total dry matter	21.20	kg/d
Daily dry matter intake	19.90	kg/d
Net energy for lactation (NE_l_)	1.75	Mcal/kg
**Production-related characteristics**		
Average milk production	37.02	kg/d
Unit of milk production balance	3.45	%
Protein digested in the small intestine when rumen-fermentable nitrogen is limiting	2.51	
Protein digested in the small intestine when rumen-fermentable energy is limiting	2.23	

DM, dry matter; TMR, total mixed ration; NE_l_, net energy for lactation. Diets were formulated according to the INRA feeding system and offered ad libitum. The TMR formulation remained unchanged throughout the study period.

**Table 17:** Internal bootstrap validation and incremental discrimination of sequential biomarker models.

Model	Apparent AUC	Bootstrap Optimism	Optimism-Corrected AUC	ΔAUC	95% CI for ΔAUC	*p*-Value
PCI	0.951	0.006	0.945	—	—	—
PCI + MDA + TAS	0.963	0.009	0.954	+0.012 †	0.003–0.021	0.009
PCI + MDA + TAS + NEFA + BCS	0.967	0.013	0.954	+0.004 ‡	−0.002–0.010	0.184

AUC, area under the receiver operating characteristic curve; PCI, Pro-inflammatory Cytokine Index; MDA, malondialdehyde; TAS, total antioxidant status; NEFA, non-esterified fatty acids; BCS, body condition score; CI, confidence interval. ΔAUC represents the difference in apparent AUC between sequential models. Pairwise AUC differences were evaluated using DeLong’s test. † Compared with PCI alone; ‡ compared with PCI + MDA + TAS.

## Data Availability

All data generated or analyzed during the study are included within the article.

## Abbreviations

The following abbreviations are used in this manuscript:

**Abbreviation**	**Definition**
ABTS	2,2′-azino-bis(3-ethylbenzothiazoline-6-sulfonic acid)
AST	Aspartate aminotransferase
AUC	Area under the receiver operating characteristic curve
BCS	Body condition score
BHBA	β-Hydroxybutyrate
BLG	β-Lactoglobulin
CD5L	CD5 molecule-like
CI	Confidence interval
CLA	Conjugated linoleic acid
COX-2	Cyclooxygenase-2
DIM	Days in milk
DM	Dry matter
ELISA	Enzyme-linked immunosorbent assay
FAME	Fatty acid methyl esters
GGT	Gamma-glutamyl transferase
GluRed	Glutathione reductase
GPx	Glutathione peroxidase
IFN-γ	Interferon gamma
IgA	Immunoglobulin A
IgG	Immunoglobulin G
IgM	Immunoglobulin M
IL	Interleukin
LF	Lactoferrin
Lp	Lactoperoxidase
LZ	Lysozyme
MCP-1/CCL2	Monocyte chemoattractant protein-1/C-C motif chemokine ligand 2
MDA	Malondialdehyde
NEFA	Non-esterified fatty acids
NEl	Net energy for lactation
NPV	Negative predictive value
OR	Odds ratio
PCI	Pro-inflammatory Cytokine Index
PPV	Positive predictive value
PTGS2	Prostaglandin-endoperoxide synthase 2
ROC	Receiver operating characteristic
RP-HPLC	Reversed-phase high-performance liquid chromatography
S100-A9	S100 calcium-binding protein A9
SCC	Somatic cell count
SD	Standard deviation
SOD	Superoxide dismutase
TAS	Total antioxidant status
TBARS	Thiobarbituric acid reactive substances
TGF-β1	Transforming growth factor beta 1
TMR	Total mixed ration
TNF-α	Tumor necrosis factor alpha
